# Stanniocalcin 2 alters PERK signalling and reduces cellular injury during cerulein induced pancreatitis in mice

**DOI:** 10.1186/1471-2121-12-17

**Published:** 2011-05-05

**Authors:** Elena N Fazio, Gabriel E DiMattia, Sami A Chadi, Kristin D Kernohan, Christopher L Pin

**Affiliations:** 1 Department of Paediatrics, The University of Western Ontario, 1151 Richmond Street, London, Ontario, N6A 3K7, Canada; 2 Department of Physiology and Pharmacology, The University of Western Ontario, 1151 Richmond St., London, Ontario, N6A 3K7, Canada; 3 Department of Biochemistry, The University of Western Ontario, 1151 Richmond St., London, Ontario, N6A 3K7, Canada; 4 Department of Oncology, The University of Western Ontario, 1151 Richmond St., London, Ontario, N6A 3K7, Canada; 5 Children's Health Research Institute, 800 Commissioners Rd. E, London, Ontario, N6C 2V5, Canada; 6 London Regional Cancer Program, 790 Commissioners Rd. E., London, Ontario, N6C 2V5, Canada

## Abstract

**Background:**

Stanniocalcin 2 (STC2) is a secreted protein activated by (PKR)-like Endoplasmic Reticulum Kinase (PERK) signalling under conditions of ER stress *in vitro*. Over-expression of STC2 in mice leads to a growth-restricted phenotype; however, the physiological function for STC2 has remained elusive. Given the relationship of STC2 to PERK signalling, the objective of this study was to examine the role of STC2 in PERK signalling *in vivo*.

**Results:**

Since PERK signalling has both physiological and pathological roles in the pancreas, STC2 expression was assessed in mouse pancreata before and after induction of injury using a cerulein-induced pancreatitis (CIP) model. Increased *Stc2 *expression was identified within four hours of initiating pancreatic injury and correlated to increased activation of PERK signalling. To determine the effect of STC2 over-expression on PERK, mice systemically expressing human STC2 (*STC2*^*Tg*^) were examined. *STC2*^*Tg *^pancreatic tissue exhibited normal pancreatic morphology, but altered activation of PERK signalling, including increases in Activating Transcription Factor (ATF) 4 accumulation and autophagy. Upon induction of pancreatic injury, *STC2*^*Tg *^mice exhibited limited increases in circulating amylase levels and increased maintenance of cellular junctions.

**Conclusions:**

This study links STC2 to the pathological activation of PERK *in vivo*, and suggests involvement of STC2 in responding to pancreatic acinar cell injury.

## Background

The stanniocalcin (STC) family of secreted glycoproteins consists of two members - STC1 and STC2 - that have 35% conserved identity and conservation of exon/intron boundaries, indicating that they are paralogs [1]. STC was first discovered in fish and shown to be involved in calcium homeostasis through effects on calcium influx [2,3]. Although terrestrial mammals rarely experience hypercalcemia, alterations in subcellular compartmentalization of Ca^2+ ^are found under pathological conditions such as the accumulation of improperly folded proteins. STC2 expression is upregulated upon induction of hypoxic or endoplasmic reticulum (ER) stress in N2a neuroblastoma cells [4]. This increase in *Stc2 *expression was reliant on the concomitant increase of Activating Transcription Factor (ATF4) [5], a protein integral to (PKR)-like endoplasmic reticulum kinase (PERK) signalling, which is part of the unfolded protein response (UPR). To date, no relationship between PERK signalling and STC2 activation has been identified *in vivo*.

The UPR is activated when ER homeostasis is disrupted. Alterations to proper functioning of ER machinery arise when protein load exceeds folding capacity, such as when luminal ER Ca^2+ ^concentrations are perturbed. The UPR consists of three signalling pathways that are individually transduced by ATF6, inositol-requiring enzyme 1 (IRE1) or PERK [6,7]. Each transducer is maintained in a repressed state by binding of the ER chaperone protein glucose regulated protein 78 (GRP78/BiP) [6,7]. When unfolded proteins accumulate, they bind to GRP78/BiP resulting in its release from the UPR transducers [8,9]. In the case of PERK, dissociation of GRP78/BiP leads to its homodimerization and autoactivation [10]. Activation of PERK results in phosphorylation of eukaryotic translation initiation factor 2α (eIF2α) [11], limiting its ability to contribute to the protein translational complex, thereby inhibiting global protein translation and alleviating the protein load on the cell [12]. Phosphorylation of eIF2α also selectively upregulates translation of mRNAs from short upstream open reading frames [13]. The predominant target mRNA for peIF2α is *Atf4*, which subsequently increases the expression of the transcriptional repressor *Atf3*, and *growth arrest and DNA damage-inducible *(*Gadd*) *34 *[14,15]. GADD34 (alternatively known as *Myd116; myeloid differentiation, primary response gene 116*, or *Ppp1r15a*; *protein phosphatase 1, regulatory [inhibitor] subunit 15A*) negatively regulates PERK signalling by combining with protein phosphatase 1 to dephosphorylate eIF2α, thereby restoring general translation [16,17].

PERK is expressed in the exocrine and endocrine pancreas under normal conditions [11,18] and *Perk*^*-/- *^mice experience deterioration of glycemic control and exocrine insufficiency over time [19]. Exocrine specific deletion of PERK revealed cellular disorganization and degranulation, increased serum amylase levels and increased cell death [20]. Our laboratory identified rapid activation of PERK signalling following induction of pancreatitis by supramaximal secretagogue stimulation and correlated decreased activation of the pathway in mice that exhibit increased sensitivity to pancreatic injury [21]. Together, these observations indicate that PERK signalling has important roles in both the physiology and pathology of the exocrine pancreas.

Given the relationship between PERK and STC2 *in vitro*, the objectives of this study were to (1) determine if a similar relationship exists in the exocrine pancreas and (2) gain insight into a role for STC2 as part of the UPR. Our results revealed a correlation of *Stc2 *expression with PERK signalling *in vivo *only after initiating pancreatic injury. Transgenic over-expression of STC2 in mice (*STC2*^*Tg*^) [22] resulted in altered PERK signalling and decreased signs of acinar cell damage associated with cerulein-induced pancreatitis (CIP). These observations suggest that STC2 is linked to PERK signalling in acinar cells and may have a role in limiting damage during pancreatic injury.

## Results

**Figure 1:**
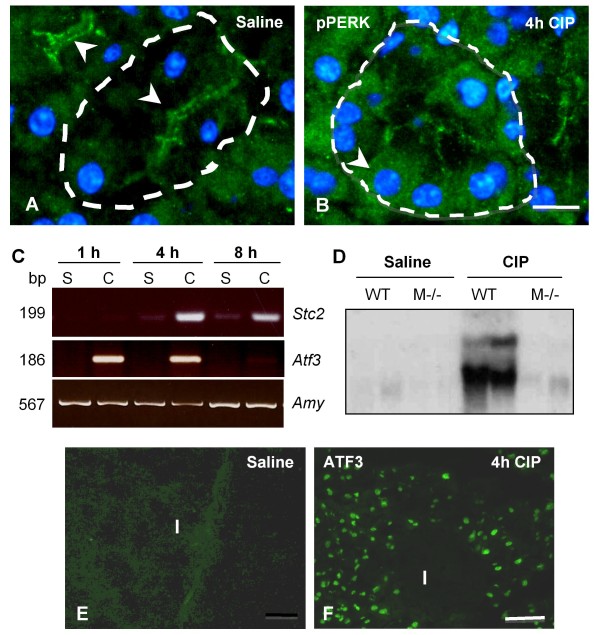
**STC2 accumulation increases in pancreatic tissue following induction of pancreatic injury**. **(A, B) **Immunofluorescence (IF) for pPERK (green) before (**A**) and 4 hours after (**B**) initiation of CIP. pPERK (arrowhead) localizes to the apical end of acinar cells under physiological conditions and more basally during CIP. Magnification bar = 13 μM. DAPI was used to counter stain nuclei. **(C) **RT-PCR of whole pancreatic RNA extracts from saline (S) and cerulein (C) treated mice showed increased accumulation of *Stc2 *and *Atf3 *mRNA after initiation of CIP in pancreatic tissue. Amplification of *Amylase *(*Amy*) was used as a positive control for RT reactions. **(D) **Northern blot analysis revealed elevated *Stc2 *mRNA four hours after initial cerulein (CIP) or saline (S) injection. No increases in *Stc2 *were observed in *Mist1*^*-/- *^(M-/-) pancreatic tissue under similar conditions. **(E, F) **IF for ATF3 expression revealed limited accumulation before CIP (**E**) and acinar-specific expression afterwards (**F**). Magnification bar = 63 μM. I = islet.

To determine if PERK signalling is active in pancreatic tissue, we performed IF with an antibody that recognizes only the phosphorylated form of PERK (pPERK). The accumulation of pPERK was restricted to a tight apical portion of the acinar cells (Figure 1A, green, arrows) typical of where Ca^2+ ^is released during the process of regulated exocytosis. RT-PCR revealed no expression of *Stc2 *in control pancreata (Figure 1C). Similar results were obtained in control pancreata for *Atf3*, another downstream target of PERK signalling suggesting that PERK activation under resting conditions does not activate all downstream mediators of PERK signalling. Our laboratory has shown that activation of PERK signalling increases during pancreatic injury [21]. To determine if the pathological activation of PERK leads to increased STC2 expression, pancreatic injury was initiated by cerulein-induced pancreatitis (CIP). Interestingly, CIP treatment led to a change in pPERK localization within four hours, with active PERK exhibiting a more diffuse and basally-restricted pattern of accumulation (Figure 1B, green, arrow), similar to the changes observed for acinar cell exocytosis under these conditions [23]. This change in localization was accompanied by increased *Stc2 *and *Atf3 *mRNA accumulation (Figure 1C). Increased *Stc2 *expression was confirmed by Northern blot analysis four hours after induction of pancreatitis (Figure 1D, saline WT vs. CIP WT). While we were unable to detect STC2 protein by IF analysis (data not shown), increased accumulation of ATF3 protein was restricted to acinar cells in CIP-treated pancreatic tissue (Figure 1E, F; I = islet). To support the link between elevated *Stc2 *levels and injury-associated PERK activation, we examined the expression of *Stc2 *in *Mist1*^*-/- *^mice, which exhibit minimal activation of PERK during injury. *Mist1 *is a transcription factor required for terminal differentiation of pancreatic acinar cells, and its absence in exocrine tissue results in improper activation of UPR components both normally and during injury [21]. In sharp contrast to wild type mice, *Stc2 *mRNA was virtually undetectable upon CIP treatment of *Mist1*^*-/- *^mice (Figure 1D; Saline *Mist1*^*-/- *^vs. CIP *Mist1*^*-/-*^).

**Figure 2:**
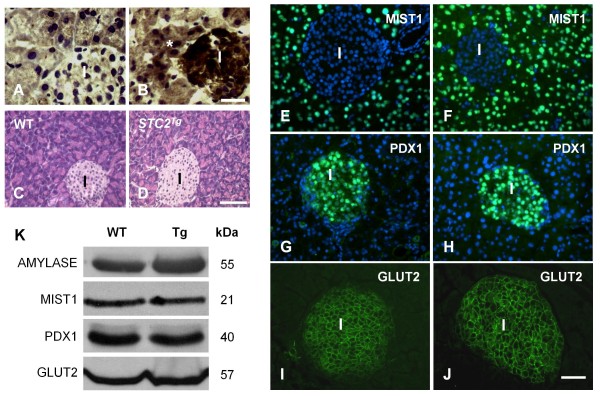
**Overall morphology and differentiation does not appear to be affected in *STC2***^***Tg***^**mice**. **(A, B) **IHC for STC2 in WT (**A**) and *STC2*^*Tg *^(**B**) pancreatic islets shows that STC2 accumulates in both acinar (*) and islet (I) tissue only in the *STC2*^*Tg *^mice. Magnification bar = 27 μm. Tissue was counterstained with hematoxylin to reveal tissue morphology. **(C, D) **Hematoxylin and eosin staining comparing general histology of pancreatic tissue in WT **(C) **or *STC2*^*Tg *^**(D) **pancreata. Magnification bar = 60 μM; I - islet. IF analysis for MIST1 **(E, F)**, PDX1 (**G, H**) or GLUT2 (**I, J**) in WT (**E, G, I**) or *STC2*^*Tg *^(**F, H, J**) pancreata. Magnification bar = 54 μm. **(K) **Representative Western blot analysis for pancreatic differentiation markers amylase, MIST1, PDX1 and GLUT2.

These findings suggest that STC2 is co-induced with PERK in acinar cells only after injury and may protect the pancreas in response to CIP. To determine if STC2 could alter the acinar cell response to pancreatic injury, we examined a mouse model in which STC2 is constitutively expressed (*STC2*^*Tg*^; [22]). IHC confirmed increased accumulation of STC2 in *STC2*^*Tg *^pancreata compared to wild type (WT) tissue, with STC2 accumulation in islets and exocrine tissue (Figure 2A, B). H&E histology (Figure 2C, D) indicated that morphology was not overtly altered in *STC2*^*Tg *^pancreatic tissue. IF staining for pancreatic differentiation markers MIST1 (exocrine, Figure 2E, F) and PDX1 (endocrine, Figure 2G, H) showed similar spatial localization and GLUT-2 accumulation was localized to the cell membrane of endocrine cells in both genotypes (Figure 2I, J). Western blot analysis confirmed equivalent levels of these markers, as well as amylase, between WT and *STC2*^*Tg *^tissue (Figure 2K).

**Figure 3:**
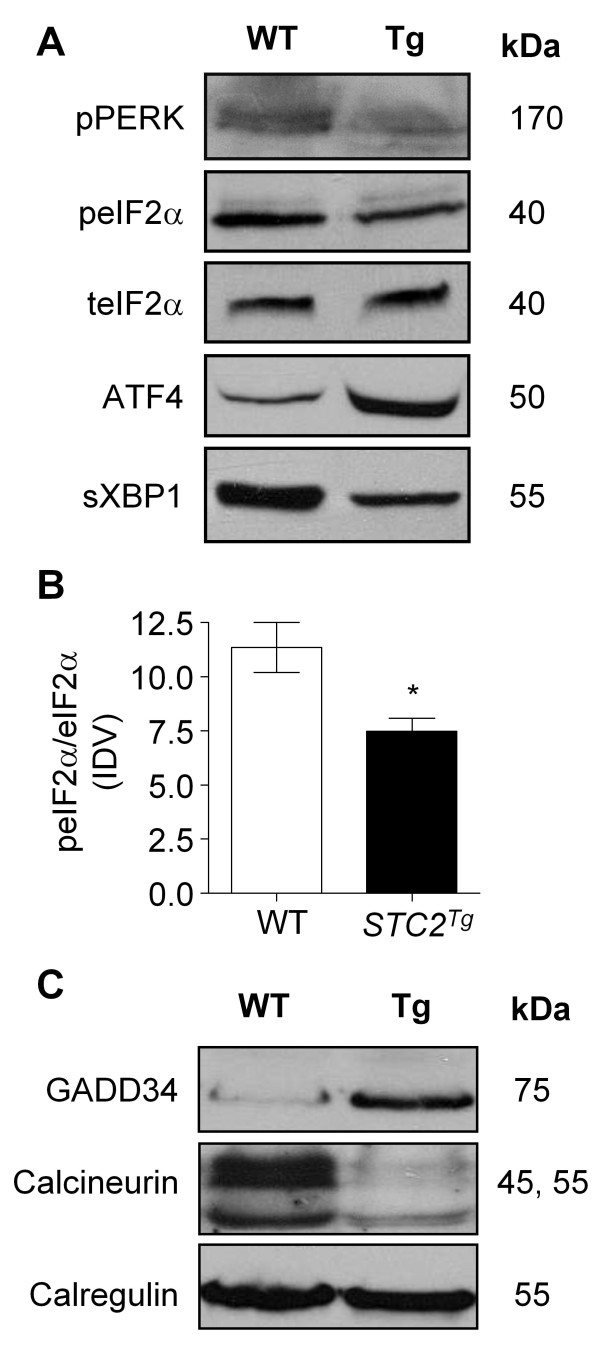
**Systemic over-expression of STC2 (*STC2***^***Tg***^**) alters the PERK signalling pathway in pancreatic tissue**. **(A) **Representative Western blot analysis for mediators of PERK signalling: pPERK, peIF2α, total (t) eIF2α, ATF4 and sXBP1 in wild type (WT) and *STC2*^*Tg *^(Tg), revealed decreased levels of pPERK, peIF2α and sXBP1, and increased amounts of ATF4 in *STC2*^*Tg *^mice. **(B) **Densitometry revealed that the ratio of peIF2α to total eIF2α is decreased in *STC2*^*Tg *^extracts relative to WT tissue (n = 3 animals; *p < 0.05). **(C) **Similar western blot analysis revealed increased accumulation of GADD34 and decreased accumulation of calcineurin in *STC2*^*Tg *^pancreatic protein extracts. No difference in calregulin accumulation was observed. Molecular weights (kDa) are provided.

We next examined PERK signalling in *STC2*^*Tg *^pancreatic tissue. PERK activation results in phosphorylation of eIF2α and translation of ATF4 [24,25]. ATF4 is required for expression of *Gadd34*, *Atf3 *and *Stc2 *[5,14,16]. Western blot analysis of WT and *STC2*^*Tg *^pancreatic tissue revealed decreased phosphorylation of both PERK and eIF2α (Figure 3A, B; n = 4 animals; p < 0.01) in *STC2*^*Tg *^tissue. Surprisingly, ATF4 accumulated to higher levels in the *STC2*^*Tg *^pancreas, suggesting dysregulation of PERK signalling. We next examined XBP1, another mediator of the UPR that is outside of the PERK signalling pathway. *Xbp1 *mRNA is targeted by the endonuclease activity of IRE1 leading to production of an active, spliced (s) form of XBP1 [26]. Western blot analysis revealed a consistent decreased in sXBP1 accumulation in *STC2*^*Tg *^mice indicating that elevated STC2 levels may also affect the other UPR pathways.

To understand why activation of PERK and eIF2α appear reduced in STC2^*Tg *^tissue, we examined regulators of these two proteins. The phosphorylation of eIF2α is reversed by GADD34 through its interaction with protein phosphatase 1 (PP1) and recent evidence indicates that calcineurin binds PERK and stimulates its autophosphorylation [27]. In *STC2*^*Tg *^pancreatic tissue, the levels of GADD34 protein were significantly higher than wild type tissue (Figure 3C). Conversely, *STC2*^*Tg *^calcineurin levels were considerably lower than WT counterparts. No changes in the ER resident protein calregulin were observed between genotypes (Figure 3C). Based on these results, it appears that over-expression of STC2 affected expression of mediators and feedback mechanisms in PERK signalling.

**Figure 4:**
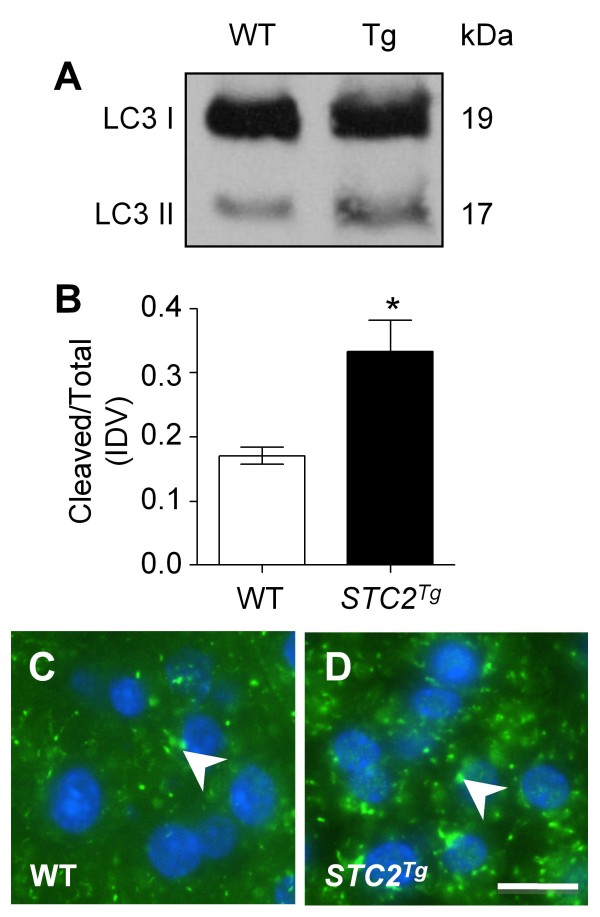
***STC2***^***Tg***^**acinar cells have increased autophagy**. **(A) **Representative Western blot analysis for LC3 I and LC3 II accumulation in wild type (WT) and *STC2*^*Tg *^(Tg) pancreatic extracts revealed increased accumulation of cleaved LC3 II which was **(B) **quantified by densitometry (n = 3 animals; *p < 0.05). IF for LC3 cellular accumulations in (**C**) WT and (D) *STC2*^*Tg *^pancreatic sections showed increased autophagic vesicles (arrowhead) in *STC2*^*Tg *^pancreatic tissue. Magnification bar = 10 μm.

Alterations to PERK/ATF4 signalling should have an effect on cell autophagy, a long-term response to ER stress, which is regulated by ATF4 during hypoxia [28]. Western blot analysis followed by densitometry revealed a significant increase in the cleaved form of the autophagy marker myosin associated protein 1 light chain 3 (LC3) in *STC2*^*Tg *^pancreatic tissue (Figure 4A, B; n = 4 animals p < 0.01) consistent with increased accumulation of ATF4 in *STC2*^*Tg *^mice. IF analysis corroborated increased autophagy as punctate LC3 staining is notably increased in *STC2*^*Tg *^exocrine tissue (Figure 4D) when compared to WT tissue (Figure 4C).

**Figure 5:**
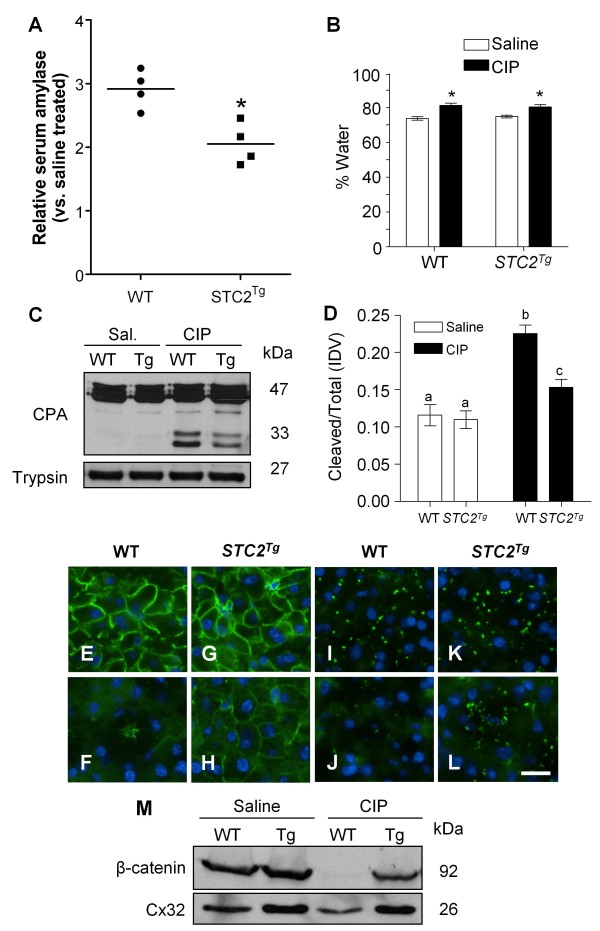
**STC2 over-expression reduces severity of acinar cell damage during cerulein-induced pancreatic injury**. **(A) **Analysis of serum amylase levels four hours into CIP treatment show that serum amylase levels rise approximately 3 fold in WT mice when compared to saline (•) whereas the increase in *STC2*^*Tg *^mice is significantly lower at 2-fold (■; * p < 0.01). **(B) **Tissue edema analysis revealed significant increases for both WT and *STC2*^*Tg *^tissue 4 hours into CIP (black bars) when compared to saline controls (white bars; n = 4 animals). No difference between genotypes was observed. **(C) **Representative Western blot for procarboxypeptidase (47 kDa) and carboxypeptidase (CPA; 33 kDa) or trypsinogen four hours after initial saline (Sal.) or cerulein (CIP) injections showed decreased accumulation of activate CPA in *STC2*^*Tg *^mice four hours into CIP. Trypsinogen levels do not differ between genotypes. **(D) **Quantification by densitometry (graph) comparing the ratio of cleaved (active; 33 kDa) to total CPA revealed a significant increase in WT tissue and, to a significantly lower extent, in *STC2*^*Tg *^tissue (n = 4 animals, letters represent statistically different values; p < 0.05). IF analysis for β-catenin **(E-H) **or Cx32 **(I-L) **in wild type (WT) or *STC2*^*Tg *^(Tg) mice four hours after initial injection of saline (**E, G, I, K**) or cerulein (**F, H, J, L**) showing decreased accumulation only in WT tissue during CIP. **(M) **Representative Western blot analysis on pancreatic protein extracts 4 hours after initial saline or cerulein (CIP) injection confirm decreased β-catenin and Cx32 accumulation only in WT-CIP treated tissue (n = 4).

The alterations in PERK signalling and cell autophagy suggested that *STC2*^*Tg *^mice should have altered sensitivity to pancreatic injury. Four hours after the induction of pancreatitis, we compared circulating serum amylase levels and tissue edema between WT and *STC2*^*Tg *^mice (Figure 5). While serum amylase levels were moderately higher in saline-treated *STC2*^*Tg *^mice, the proportional increase in serum amylase levels following CIP was significantly lower in *STC2*^*Tg *^mice when compared to WT mice, indicative of decreased sensitivity to pancreatic insult (Figure 5A; n = 4 animals; p < 0.05). However, no difference was observed in tissue edema after CIP between genotypes (Figure 5B). We next examined the effects of pancreatic injury at the cellular level by examining enzyme activation and cell structures. Western blot analysis of procarboxypeptidase (CPA) activation revealed reduced levels of active CPA in CIP *STC2*^*Tg *^pancreatic tissue when compared to WT cerulein-treated tissue (Figure 5C, D). Interestingly, analysis of cell junction proteins showed continued expression in *STC2*^*Tg *^tissue following CIP that was not evident in WT tissue. In WT acinar cells, the expression of β-catenin decreased to negligible levels, indicative of a loss in adherens junctions between acinar cells as expected (Figure 5E, F). However, β-catenin remained readily detectable in *STC2*^*Tg *^pancreatic tissue after induction of CIP (Figure 5G, H). Similarly, punctate connexin32 (Cx32) accumulation was lost in WT (Figure 5I, J) but not in *STC2*^*Tg *^acini (Figure 5K, L) suggesting a greater disruption in gap junction complexes in WT acinar cells. Western blot analysis corroborated IF data with CIP leading to a more dramatic loss of β-catenin and Cx32 accumulation in WT extracts compared to *STC2*^*Tg *^extracts after four hours (Figure 5M) indicating that intercellular complexes were protected in *STC2*^*Tg *^mice, suggestive of reduced cellular damage. Combined, these findings suggest that acinar cell integrity is maintained to a higher degree when acini are exposed to higher than normal levels of STC2.

**Figure 6:**
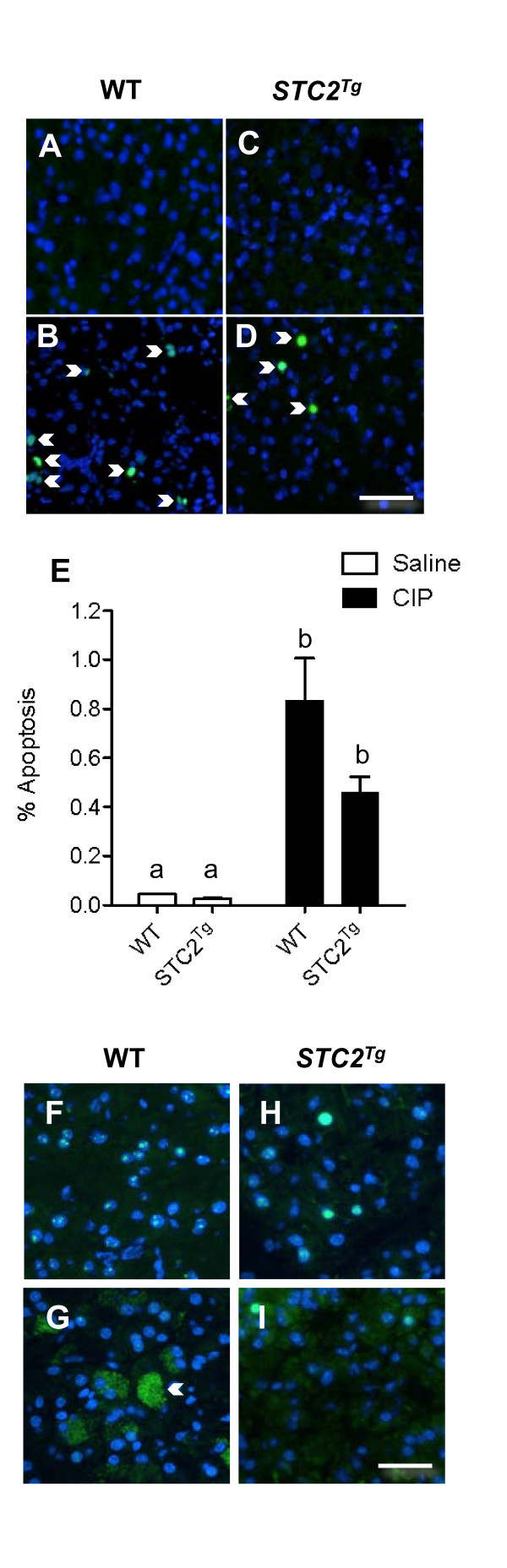
**Cell death is decreased during CIP in *STC2***^***Tg***^**tissue**. TUNEL analysis revealed apoptosis (arrowheads) during CIP (**B, D**) but not saline injections (**A, C**) in WT (**A**, **B**) and *STC2*^*Tg *^(**C**, **D**) pancreata. Magnification bar = 45 μM. **(E) **Quantification of TUNEL positive cells in WT and *STC2*^*Tg *^tissue (n = 4 animals; letters indicate significant differences). **(F-I) **HMGB1 IF to identify necrosis revealed nuclear localization in saline conditions for WT (**F**; n = 3 animals) and *STC2*^*Tg *^(**H**; n = 3 animals) tissue. After induction of pancreatitis, patches of cells with cytoplasmic, punctate HMGB1staining, indicative of necrosis, were observed in WT tissue (**G**; n = 4 animals) but not in *STC2*^*Tg *^tissue (**I**; n = 4 animals). Magnification bar = 34 μM

As a final marker for CIP severity, we assessed the amount of acinar cell necrosis and apoptosis within the two genotypes. The ratio of necrosis to apoptosis correlates to CIP severity [29]. Visualization of apoptosis using TUNEL staining revealed increased apoptosis in both WT (Figure 6A, B; arrowheads) and *STC2*^*Tg *^(Figure 6C, D; arrowheads) pancreatic tissue after induction of pancreatitis. Quantification of TUNEL positive cells revealed slight decreases in acinar cell apoptosis in *STC2*^*Tg *^tissue (Figure 6E). We next examined necrosis through IF for High mobility group protein b1 (HMGB1), a protein that interacts with DNA, but upon necrotic stimulus, is released from the nucleus and the cell to participate in inflammatory responses [30,31]. Cytoplasmic staining of HMGB1 is routinely used as an indicator of cell necrosis. HMGB1 is localized to the nucleus in both WT (Figure 6F) and *STC2*^*Tg *^(Figure 6H) saline treated tissue. After induction of pancreatitis, punctate, cytoplasmic HMBG1 staining was observed in distinct patches of cells only in WT tissue (Figure 6G). All *STC2*^*Tg *^tissue analysed was devoid of punctate cytoplasmic HMGB1 staining (Figure 6I). This analysis reveals that the levels of both apoptosis and necrosis are decreased in *STC2*^*Tg *^tissue, indicative of less tissue damage after CIP.

## Discussion

STC2 is a secreted protein that, when expressed to high levels in mice, has profound effects leading to growth restriction [22]. However, ablation of *Stc2 *in mice results in limited phenotypic alterations suggesting that STC2 functions may fall outside normal physiology [32]. In support of such a hypothesis, *in vitro *analysis has identified increased *Stc2 *accumulation following treatment with thapsigargin or tunicamycin, agents that activate ER stress pathways [5]. STC2 is also up-regulated in response to hypoxic insult and is expressed to higher levels in many tumor-derived cell lines [4,33]. This suggests that STC2 may be involved in responding to external challenges that activate cell stress pathways. In this study, we have identified increased *Stc2 *accumulation within four hours of inducing pancreatic injury that correlated with activation of the PERK signalling. Importantly, forced expression of STC2 in mice altered PERK signalling and reduced cellular damage in response to pancreatic injury. These results suggest a role for STC2 responding to and mediating the effects of cell stress in pancreatic tissue.

Studies assessing the expression of *Stc2 *have revealed a limited expression pattern in adult tissues. In the human pancreas, *Stc2 *is specifically expressed in alpha cells of the islets of Langerhans and not in acinar tissue [1]. Our mRNA analysis revealed murine pancreatic expression of *Stc2 *only after induction of pancreatitis by supramaximal secretagogue stimulation. Unfortunately, IHC on CIP-treated tissue sections did not detect STC2 protein (data not shown) indicating that STC2 may be expressed at lower than detectable levels or that it is rapidly secreted into the circulation. While we have not definitively identified acini as the origin of *Stc2 *expression, *Atf3*, a downstream mediator of PERK signalling, accumulated only in acinar cells during CIP, suggesting that CIP treatment leads to a cell autonomous activation of *Stc2*. As observed in HEK293 and N2a neuroblastoma cells, the increased accumulation of *Stc2 *is consistent with activation of PERK signalling in response to injury [5]. Induction of *Stc2 *mRNA levels was not observed after CIP in *Mist1*^*-/- *^mice, which do not activate PERK or ATF3, and experience increased severity of CIP [21]. These findings support the activation of *Stc2 *as a protective mechanism, potentially as a mediator of the UPR.

While previous studies have correlated increased expression of STC2 as part of the UPR, the effects of STC2 on PERK signalling have not been examined. We have now shown that systemically high levels of STC2 reduce PERK activation. *STC2*^*Tg *^mice showed decreased phosphorylation of PERK and eIF2α in pancreatic tissue suggesting that continued exposure to STC2 may lead to activation of negative feedback mechanisms. Since no change in total eIF2α is observed, this difference is peIF2α is specific to the phosphorylation event and not decreased eIF2α accumulation. While we showed a decrease in pPERK, total PERK levels were not assessed and this could account for possible differences in activation of the pathways as well.

We have identified at least two effectors of the PERK signalling pathway that were changed in *STC2*^*Tg *^mice. First, GADD34, which combines with protein phosphatase 1 to reduce eIF2α phosphorylation [17], accumulated to higher levels. Second, we observed significantly decreased accumulation of calcineurin, which stimulates PERK autophosphorylation, thereby increasing activity [27]. Whether these changes are reflective of a direct role for STC2 in their regulation or a response to other events caused by over exposure to STC2 is unclear. However, the altered expression of these factors suggests a role for STC2 in regulating Ca^2+ ^homeostasis since both calcineurin and PERK are affected by altered Ca^2+ ^sequestration in the ER. These data suggest that constitutive exposure to STC2 results in depression of the activity of key factors regulating the UPR.

In light of decreased PERK and eIF2α activation, it was surprising that increased accumulation of ATF4 was observed in the pancreas of *STC2*^*Tg *^mice. Phosphorylation of eIF2α is known to stimulate translation of *Atf4 *mRNA, leading to increased ATF4 protein accumulation. However, increased ATF4 expression, and the subsequent increase in GADD34 could be mediated by increased stability of the ATF4 protein. Recent studies have shown that ATF4 is stabilized by interaction with p300 through inhibition of ubiquitination [34], and that association with p300/CBP enhances ATF4 transcriptional ability [34,35]. It is possible that in *STC2*^*Tg *^mice, stabilization of ATF4 is enhanced regardless of decreased phosphorylation of eIF2α, accounting for its increased expression. Although there is little known about non-peIF2α related transcriptional activation of ATF4, increased transcriptional activation by a yet to be identified factor cannot be ruled out.

**Figure 7:**
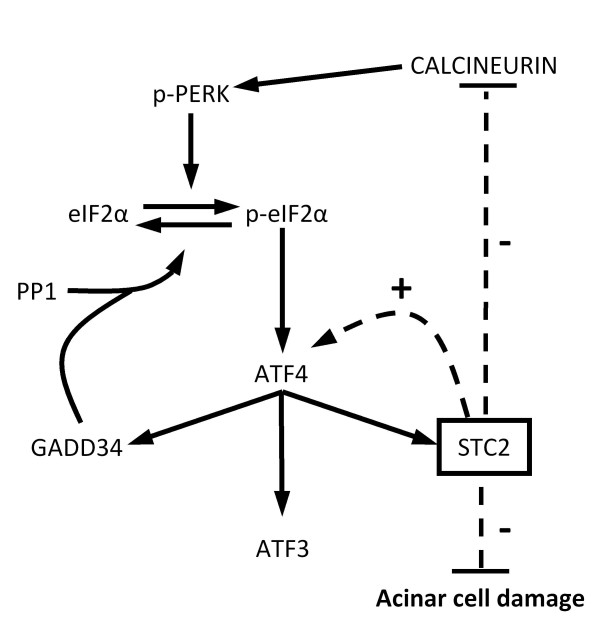
**Schematic pathway showing a possible role for STC2 in affecting the PERK signalling pathway**. The increased accumulation of ATF4 leads to increased expression of GADD34, ATF3 and STC2. Elevated levels of STC2 can alter both PERK phosphorylation and ATF4 levels through as yet undetermined mechanisms (dashed arrows), as well as promote reduced damage during pancreatic injury. Whether this protective effect of STC2 is a normal function of the protein needs to be clarified.

Figure 7 summarizes a potential role for STC2 in the UPR, where over-expression of STC2 negatively affects calcineurin expression, (Figure 7, dotted line), which would lead to inhibition of calcineurin-dependent phosphorylation of PERK. Decreased phosphorylation of PERK results in decreased phosphorylation of eIF2α, as observed in *STC2*^*Tg *^pancreatic tissue. Although the increased expression of ATF4 in *STC2*^*Tg *^tissue is not intuitive considering the phosphorylation of both PERK and eIF2α, it reveals a potential role for STC2 in ATF4 regulation (Figure 7, dotted arrow). This increase in ATF4 expression leads to increased GADD34 expression, which acts as a negative feedback mechanism to contribute to decreased phosphorylation of eIF2α [16].

As would be expected from increased ATF4 expression, *STC2*^*Tg *^mice also exhibited an increased level of autophagy, a downstream consequence of elevated ATF4 levels. Autophagy is important for maintenance of cellular homeostasis through balancing synthesis and degradation or recycling of cellular components. Independent studies have indicated that either phosphorylation of eIF2α or expression of ATF4 is required for induction of autophagy *in vitro *[28,36,37]. The increased expression of ATF4 in *STC2*^*Tg *^pancreatic tissue may be the cause of increased autophagy. Interestingly, we did not observe increased acinar cell apoptosis (data not shown), which is another downstream result of increased UPR. Therefore, it appears that only certain parts of the UPR have been activated in *STC2*^*Tg *^mice.

Whether STC2 is protective in nature is still unclear since studies have shown it has both protective [38] and detrimental roles [4,33] in cancer progression *in vitro*. Our results suggest a protective advantage following exocrine pancreatic injury *in vivo*. *STC2*^*Tg *^mice exhibit a decreased proportional increase in serum amylase levels, increased maintenance of cellular junctions, decreased apoptosis and necrosis and decreased activation of CPA in exocrine pancreatic tissue, all suggestive of decreased pancreatitis severity. In addition, the enhanced autophagy observed in *STC2*^*Tg *^acinar tissue would facilitate more rapid degradation of resident digestive enzymes upon injury, thereby limiting damage to cellular contents. This would account for increased maintenance of cellular junctions. However, to truly understand the role of STC2 in pancreatic injury, further experiments in mice lacking *Stc2 *mice should be performed.

## Conclusions

In conclusion, this is the first study to correlate PERK signalling and STC2 *in vivo*, and suggests two novel roles for STC2, as a downstream effector of PERK signalling and possible protective factor during pancreatic injury. This is also the first time that STC2 has been shown to affect proteins that regulate Ca^2+^-mediated response in the cell.

## Methods

### Mouse handling

For characterization of *Stc2 *expression during cerulein-induced pancreatitis (CIP), wild type and *Mist1*^*-/- *^mice were maintained on a C57/Bl6 background. For analysis of pancreatitis severity, *STC2*^*Tg *^and wild type mice are maintained on a C57/Bl6 × CBA background. Mice carrying a targeted ablation of the *Mist1 *gene (*Mist1*^*-/-*^) [39] and transgenic mice that express *STC2 *(*STC2*^*Tg*^) from the *CMV *promoter + chicken *β-actin *promoter [22], have previously been described. All experiments were approved by the Animal Care Committee at the University of Western Ontario (Protocol #116-2008) and mice handled according to regulations stipulated by the Canadian Council on Animal Care.

### Induction of pancreatitis

To induce pancreatic injury, 2-4 month-old female mice were given up to seven hourly intraperitoneal injections of cerulein (50 μg/kg body weight; Sigma-Aldrich). As a control, mice were injected with 0.9% saline. Mice were sacrificed 1, 4 or 8 hours after initial cerulein injection and pancreatic tissue from each was immediately processed to isolate RNA, protein, and tissue sections for histological or immunohistochemical analysis. To assess serum amylase, blood was obtained via cardiac puncture, placed on ice for 20 minutes and centrifuged at 4°C, 2500 rpm for 15 minutes. Serum amylase was quantified using a Phadebas amylase assay (Magle Life Sciences, Lund, Sweden) as per manufacturer's instructions.

### Tissue preparation and histology

Pancreatic tissue was either directly embedded in OCT or incubated in formalin for 24 hours followed by paraffin embedding. For morphological analysis, paraffin embedded tissue was sectioned to 6 μM and stained with hematoxylin and eosin. Immunofluorescent (IF) analysis was performed on fresh frozen sections as described previously [40]. Primary antibodies and their dilutions are listed in Table 1. Following primary antibody incubation and PBS washes, sections were incubated for 1 hour with the secondary antibodies of either anti-rabbit FITC or anti-mouse TRITC (1:250; Sigma-Aldrich). After further washing, sections were incubated with 4',6-diamidino-2-phenylindole (DAPI; 1:1000) and slides were mounted with Vectashield (Vector Laboratories, Burlingame CA, USA). Histological staining was visualized with a Leica DMIOS upright microscope and images were captured using OpenLab 4.0.3 Software (PerkinElmer, Waltham, MA, USA).

**Table 1:** List of Primary Antibodies Used in Fazio et al (2011)

Antigen	Species	Company	Dilution	Method	Reference
ATF3	Rabbit	Santa Cruz Biotechnology (Santa Cruz, CA, USA)	1:1000	IB/IF	Wu *et al. *(2010). Nature. 465: 368-372
ATF4	Rabbit	Santa Cruz Biotechnology	1:500	IB	Matsushita *et al. *(2009). Mol. Cell. Biol. 29: 5843-5857.
Calcineurin	Mouse	Sigma (St. Louis, MO, USA)	1:1000	IB	
Calregulin	Rabbit	Santa Cruz Biotechnology	1:1000	IB	
Pro-CPA	Rabbit	AbD Serotec (Oxford, UK)	1:2000	IB	
β-catenin	Mouse	Transduction Laboratories (San Jose, CA, USA)	1:1000	IB/IF	
Connexin 32	Mouse	Millipore (Temecula, CA, USA)	1:1000	IB/IF	
peIF2α	Rabbit	Invitrogen (Carlsbad, CA, USA)	1:1000	IB	Harding *et al. *(2009). *PNAS*. 106(6), 1832-1837
total eIF2α	Rabbit	Cell Signalling (Beverly, MA, USA)	1:1000	IB	Nanbo *et al. *(2002) *EMBO J. *21, 954-965.
GADD34	Rabbit	Santa Cruz Biotechnology	1:500	IB	Makris *et al. *(2000). Mol. Cell. 5: 969-979.
GLUT2	Rabbit	Millipore	1:1000	IB/IF	
HMGB1	Rabbit	Novus Biologicals (Littleton, CO, USA)	1:500	IF	
LC3	Rabbit	Novus Biologicals	1:1000	IB/IF	
MIST1	Rabbit	ProSci Incorporated (Poway, CA, USA)	1:500	IB/IF	
PDX1	Rabbit	Abcam (Cambridge, MA, USA)	1:1000	IB/IF	
pPERK	Rabbit	Santa Cruz Biotechnology	1:500	IB/IF	Denis *et al. *(2010). Neuroscience. 170(4):1035-44
Trypsin	Rabbit	Chemicon (Temecula, CA, USA)	1:2000	IB	

1 - Abbreviations; IB, immunoblotting; IF, Immunofluorescence

### TUNEL analysis

TUNEL analysis was used to identify apoptotic cells using the *In situ *Cell Death Detection kit (Roche, Laval, QC, Canada) following manufacturer's instructions. Briefly, frozen sections were fixed, washed and permeabilized before labelling with TUNEL reaction mixture for 60 minutes. After washing, sections were counterstained with DAPI, coverslipped and visualized using a Leica DMIOS upright microscope and images were captured using OpenLab 4.0.3 Software (PerkinElmer, Waltham, MA, USA). Analysis of percent apoptotic cells was carried out by comparing TUNEL positive cells to total cell number. Cell counts based on DAPI were done using Image J software, and TUNEL positive cells were determined manually. Ten random fields of view from 4 sections per animal were used for analysis.

### RNA isolation, Northern blotting and Real-Time RT-PCR

RNA was isolated from the splenic portion of the pancreas and processed using TRIzol (Invitrogen, Burlington, ON, Canada) as described [21]. Northern blotting was performed as previously described [39]. Thirty μg of RNA was resolved by electrophoresis on a 1% agarose/formaldehyde gel and blotted onto Hybond membrane (GE Healthcare, Baie d'Urfe, QC, Canada). Membranes were hybridized overnight at 42°C with an α-^32^PdCTP radiolabelled probe for *Stc2 *or for *18S *rRNA as a loading control.

For RT-PCR, two μg of RNA was reverse transcribed using Improm-II reverse transcriptase and random primers (Promega, Madison WI, USA). RT-PCR for *Stc2*, *Atf3*, *amylase *and *β-actin *was performed with 1 μL of cDNA and *Taq *DNA polymerase (Promega, Madison WI, USA).

### Protein isolation and immunoblot analysis

Protein extraction was carried out to obtain both nuclear and cytoplasmic fractions. Briefly, 5 volumes of buffer CE pH 7.6 (10 mm Hepes, 60 mM KCl, 1 mM EDTA, 0.075% v/v NP-40, 1 mM DTT, 1 mM PMSF) were added to whole pancreatic tissue prior to homogenization. Homogenized cells were centrifuged at 1000 rpm, 4°C for 4 minutes. The supernatant was removed and saved as cytoplasmic extract. The nuclear pellet was gently washed with CE buffer lacking NP-40 and centrifuged again. After removal of the supernatant, the nuclear pellet was covered with 1 pellet volume of NE buffer (20 mm Tris-HCl, 420 mM NaCl, 1.5 mm MgCl_2_, 0.2 mM EDTA, 1 mM PMSF, 25% w/v glycerol) and the NaCl concentration was adjusted to 400 mM for the entire volume. An additional pellet volume of NE buffer was added followed by vortexing for resuspension. The extract was incubated on ice for 10 minutes with intermittent vortexing. Both cytoplasmic and nuclear extracts were centrifuged at 14,000 rpm, 4°C, for 10 minutes to pellet any debris. Nuclear extracts were used for analysis of MIST1, PDX1, XBP1 and ATF4 levels, whereas cytoplasmic extracts were used for all other immunoblot analysis.

Electrophoresis and immunoblotting were carried out as previously described [41]. For immunoblot analysis, 40 to 60 μg of pancreatic protein was resolved by SDS/PAGE and transferred to PVDF membrane (Biorad, Mississauga, ON, Canada). Blots were blocked with 5% non-fat dry milk (NFDM) and probed with primary antibodies diluted in 5% NFDM overnight at 4°C. For primary antibodies and their dilutions, see Table 1. Blots were washed three times in PBS supplemented with Tween-20 (PBS-T) then incubated with anti-rabbit or anti-mouse horseradish peroxidase conjugated secondary antibody (1:10,000 or 1:2000, Jackson Labs, Bar Harbor, ME, USA) in 5% NFDM for one hour. Blots were subjected to another series of washes in PBS-T, incubated with Western Lightning chemiluminescence substrate (Perkin Elmer, Waltham MA, USA), exposed to X-ray film (Fisher Scientific, Ottawa, ON, Canada) and developed. Denistometry was performed on immunoblot autoradiographs by analysis with a FluorChem 8800 documentation system and accompanying FluorChem 8800 software (Alpha Innotech, San Leandro CA, USA).

### Statistical Analyses

All statistical analyses were performed using Graphpad Prism 4.02 (Graphpad Software, San Diego CA, USA). Carboxypeptidase cleavage and serum amylase levels were analyzed using a two-way ANOVA followed by a Bonferroni post-hoc test. Levels of phosphorylated eIF2α and LC3 cleavage were statistically analyzed using a Mann-Whitney test.

## Authors' contributions

EF carried out the animal studies, protein and gene expression analysis, performed the statistical analysis and drafted the manuscript. KK carried out some immunofluorescent analysis and SC performed Northern blot analysis. GD provided mouse lines and participated in its design and coordination and helped draft the manuscript. CP conceived the study, and participated in its design and coordination and helped to draft the manuscript. All authors read and approved the final manuscript.
